# Effect of Various Carbon Electrodes on MIP-Based Sensing Proteins Using Poly(Scopoletin): A Case Study of Ferritin

**DOI:** 10.3390/biomimetics9070426

**Published:** 2024-07-13

**Authors:** Aysu Yarman

**Affiliations:** Molecular Biotechnology, Faculty of Science, Turkish-German University, Istanbul 34820, Türkiye; yarmanl@tau.edu.tr or yarman@tau.edu.tr

**Keywords:** ferritin, molecularly imprinted polymers, biomimetic sensors, screen-printed electrodes, glassy carbon electrodes

## Abstract

Sensitivity in the sub-nanomolar concentration region is required to determine important protein biomarkers, e.g., ferritin. As a prerequisite for high sensitivity, in this paper, the affinity of the functional monomer to the macromolecular target ferritin in solution was compared with the value for the respective molecularly imprinted polymer (MIP)-based electrodes, and the influence of various surface modifications of the electrode was investigated. The analytical performance of ferritin sensing was investigated using three different carbon electrodes (screen-printed carbon electrodes, single-walled-carbon-nanotube-modified screen-printed carbon electrodes, and glassy carbon electrodes) covered with a scopoletin-based MIP layer. Regardless of the electrode type, the template molecule ferritin was mixed with the functional monomer scopoletin, and electropolymerization was conducted using multistep amperometry. All stages of MIP preparation were followed by evaluating the diffusional permeability of the redox marker ferricyanide/ferrocyanide through the polymer layer by differential pulse voltammetry. The best results were obtained with glassy carbon electrodes. The MIP sensor responded up to 0.5 µM linearly with a K_d_ of 0.30 µM. Similar results were also obtained in solution upon the interaction of scopoletin and ferritin using fluorescence spectroscopy, resulting in the quenching of the scopoletin signal, with a calculated K_d_ of 0.81 µM. Moreover, the binding of 1 µM ferritin led to 49.6% suppression, whereas human serum albumin caused 8.6% suppression.

## 1. Introduction

Nature needed millions of years for the evolution of proteins and nucleic acids, which exhibit high affinity and selectivity in binding to or chemical conversion of different partners. Nowadays, biomimetic binders and catalysts can be generated within several days using “evolution in the test tube” of non-natural nucleotides or total chemical synthesis of (molecularly imprinted) polymers [1]. According to the International Union of Pure and Applied Chemistry (IUPAC), “biomimetic” refers to a laboratory method designed to replicate a natural chemical process or a compound that mimics the structure or function of a biological material. The lotus effect, observed in water-repellent surfaces, is a prominent example of a biomimetic system.

In recent years, there has been a tremendous interest in substituting biochemical reagents in bioanalysis and separation techniques with fully synthetic organic polymers, specifically molecularly imprinted polymers (MIPs, the so-called artificial antibodies) [1]. In analogy to the binding of antigens and haptens in the antigen-binding sites, the paratope of antibodies, recognition by MIPs is also based on the structural complementarity of the binding pocket and target. MIPs, in particular, have gained attention for their versatile applications in biotechnology. These include their successful use in solid-phase extraction and chromatography, in replacing antibodies in ELISAs, and as sensors for low-molecular-weight substances and biomacromolecules [2,3,4,5,6,7,8,9,10,11,12,13,14,15,16,17,18,19,20,21]. 

**Scheme 1:**
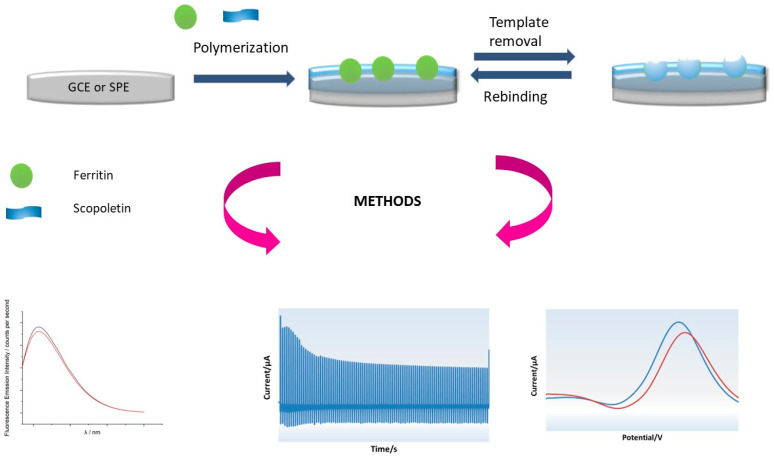
Schematic representation of the preparation and characterization of the ferritin–MIP sensor. GCE: glassy carbon electrode; SPE: screen-printed electrode (Blue curve: Without ferritin and Red Curve: With ferritin).

The synthesis of MIPs, pioneered by Wulff and Mosbach, involves polymerizing functional monomers (with or without cross-linkers) in the presence of a target analyte, known as the template [22,23]. Removal of the template from the polymer results in the formation of binding cavities with a molecular memory of the template’s size, shape, and functionality (Scheme 1). Unlike enzymes and antibodies, which are built from 20 natural amino acids, MIPs can be prepared using one to six functional monomers, even without a cross-linker. Furthermore, MIPs exhibit higher stability under harsh conditions (e.g., pH, temperature, organic solvents) and are easier to regenerate compared to their biological counterparts [1,24].

A wide range of MIPs have been developed to recognize various targets, especially low-molecular-weight ones, with some already commercially available. However, the development of MIPs for biomacromolecules has been more limited, constituting only around 10% of annually published papers on MIPs [7]. The scarcity of MIPs for high-molecular-weight compounds is largely attributed to stability issues of biomacromolecular templates in the polymerization media used in conventional bulk imprinting processes [7,25].

Another challenge of the traditional bulk imprinting process is the potential entrapment of template molecules within the polymer matrix, hindering their removal and subsequent rebinding. Surface imprinting techniques, including electropolymerization, have partially addressed these limitations [7,26,27,28,29,30]. Electropolymerization enables polymer synthesis under mild conditions with adjustable thickness. Moreover, polymer films can be directly formed on the transducer, eliminating the need for additional immobilization steps. Different types of metal and carbon electrodes have been applied in order to optimize the analytical performance, especially sensitivity [1]. Commonly used electroactive monomers include pyrrole, *o*-phenylenediamine (*o*-PD), aniline, scopoletin, *p*-aminophenylboronic acid, phenol, and thiophene and their derivatives [2,7,14].

The scopoletin molecule offers effective aromatic π–π interactions and hydrogen bonding for the “pre-polymerization complex” with different template molecules. The aromatic system of scopoletin exhibits fluorescence behavior, and quenching resulting from interaction with the respective target analyte has been applied to determine the affinity expressed by the dissociation constant K_d_ in solution [31]. Polymerization of the natural coumarin derivative scopoletin proceeds at moderate anodic potentials and does not require deoxygenation of the monomer solution. It yields a non-conductive, conformal, hydrophilic film on different electrode materials [7]. These ultra-thin films, in the nanometer range, only partially entrap the protein template, thus enabling the effective removal of the template from the MIP and fast rebinding. For the electrochemical readout, either the direct electron transfer of redox-active proteins, including ferritin, or the modulation of permeability for redox markers by protein binding (“redox gating”) has been applied. The spontaneous adsorption of the protein target at the electrode surface and changes in the polymer structure on changing the background solution will overlay the signal of redox gating for the binding of the target to the MIP [32]. After polymerization, electrostatic interactions are involved in the interaction with the target because the poly(scopoletin) film on Au electrodes has a negative zeta potential [33]. This polarization should suppress the non-specific binding of negatively charged substances, including ferritin (IP 4.52).

This work investigates the analytical performance of ferritin sensing using three different carbon electrodes (screen-printed carbon electrodes, single-walled-carbon-nanotube-modified screen-printed carbon electrodes, and glassy carbon electrodes) covered with a scopoletin-based MIP layer. Ferritin, the central iron storage protein in human iron metabolism, is a biomarker for iron deficiency, cell damage, and inflammation [34]. It has a globular structure with a molecular weight of 450 kDa and a diameter of 12 nm. It also possesses an isoelectric point of 4.52. Various strategies, including immunoassays (enzyme-linked immunosorbent assay [ELISA] and radioimmunoassay) and spectroscopic methods (colorimetry, luminescence measurement, nephelometry, turbidimetry, etc.), have been developed for its analysis [35]. In addition to its function as a biomarker, it is an interesting target for MIP research because its diameter is close to that of colloids and viruses. Thus, the achievements can be transferred to developing imprinted polymers for these bio-particles [36].

Regardless of the electrode type, a simple electrode preparation method, namely random imprinting, was used. As illustrated in the scheme, the template molecule ferritin was mixed with the functional monomer scopoletin, and electropolymerization was conducted using pulse amperometry (Scheme 1) to create films with good adhesion to the electrode surface [7]. The application of pulsed potentials also supports the replenishment of the depleted layer adjacent to the MIP surface by the low diffusion of the macromolecular protein and enhances the incorporation of the template into the polymer. The film thickness can be precisely adjusted by the charge consumed during electropolymerization [7]. Various strategies were used to remove the template molecules from the polymer film. Electrochemistry, mainly differential pulse voltammetry, was used to characterize all stages of MIP preparation. Furthermore, the affinity of the functional monomer to the macromolecular target ferritin in solution was compared with the value for the respective MIP, and the influence of various surface modifications of the electrode was investigated.

Achieving sensitivity in the sub-nanomolar concentration range is essential for the accurate determination of critical protein biomarkers, such as ferritin. A ferritin concentration above 150 µg/L in menstruating women and 200 µg/L in men and non-menstruating women who are otherwise healthy may suggest a risk of iron overload (conditional recommendation based on prior WHO guidelines). In adults with underlying health conditions, a ferritin level exceeding 500 µg/L may also indicate a potential risk of iron overload [37].

## 2. Materials and Methods

### 2.1. Chemicals and Reagents

Scopoletin (7-hydroxy-6-methoxycoumarin), ferritin from the equine spleen, human serum albumin (HSA), and HEPES were purchased from Sigma-Aldrich (Steinheim, Germany). Ferricyanide, ferrocyanide, and sodium chloride were supplied by MERCK KGaA (Darmstadt, Germany). All reagents were of analytical grade and used without further purification.

### 2.2. Preparation of Ferritin-Imprinted Sensors and Measurements

Glassy carbon disk electrodes (3 mm in diameter; CH Instruments, Austin, TX, USA), screen-printed carbon electrodes (4 mm in diameter; SPC from DropSens, Oviedo, Spain), and single-walled-carbon-nanotube-modified screen-printed carbon electrodes (4 mm in diameter; SWSP from DropSens, Oviedo, Spain) were used for MIP synthesis and for voltammetric measurements (PalmSens potentiostat, Utrecht, The Netherlands).

Before electropolymerization, the GCEs were cleaned with 30% nitric acid for 15 min. Afterward, mechanical cleaning was performed with 1.0 μm, 0.3 μm, and 0.05 μm of alumina slurry. The electrodes were rinsed with ethanol and Millipore water by ultrasonication. The screen-printed electrodes were activated in 0.1 M H_2_SO_4_ for 2 min at 3 mA using chronopotentiometry.

Based on previous experiences and the suggestion in Malitesta’s recent paper, electropolymerization on the GCEs and screen-printed electrodes was performed in a mixture of 0.5 mM scopoletin (10 mM stock solution in ethanol), 100 mM NaCl (1 M stock in water), and 10 µM ferritin (100 µM stock solution in 10 mM HEPES buffer, pH 7.4) solutions [38,39]. End concentrations in the mixture were obtained by dilution with water. Multistep amperometry was applied, involving 100 cycles, in which each GCE (or SPE) was polarized at 0 V for 1 s and at 0.9 V for 0.1 s. Various strategies, such as electrochemical removal and incubation in alkaline, acidic, or salt solutions, were used to remove the template molecules from the polymer film.

The rebinding and removal of ferritin were followed by changes in DPV responses of 5 mM [Fe(CN)_6_]^3−/4–^ solution (in 100 mM KCl). This was achieved by applying a potential range of −0.2 to 0.7 V, a potential step of 5 mV, a pulse amplitude of 25 mV, and a pulse duration of 0.7 s. Moreover, cyclic voltammetry was applied to follow the fabrication steps. The potential was swept between −0.2 and 0.7 V, with a scan rate of 50 mV/s (3 scans).

Fluorescence measurements were performed using the M-4/2003 Quanta Master Steady State Spectrofluorometer (Photon Technology International, London, ON, Canada) as follows: The concentration of ferritin (stock solution: 1 mM in 10 mM HEPES buffer, pH 7.4) was varied in 0.5 mM scopoletin solution containing 100 mM NaCl. As ferritin was incrementally added, from 0 to 25 µM, the fluorescence emission of scopoletin (with an excitation wavelength of 430 nm and an emission wavelength of 460 nm) exhibited partial quenching [31]. The spectral alterations corresponding to different ferritin concentrations were analyzed using the One-Site-Specific Binding Model (y = B_max_ × x/(K_d_ + x) to determine the dissociation constant governing the interaction between ferritin and scopoletin (B_max_: maximum response; and K_d_: dissociation constant).

## 3. Results and Discussion

### 3.1. Screen-Printed Electrodes

#### 3.1.1. Screen-Printed Carbon Electrodes

**Figure 1:**
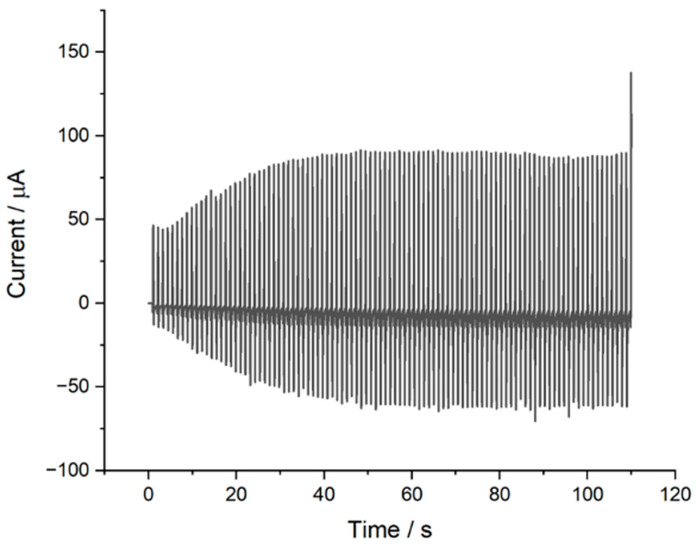
MIP film formation showing current–time curves for 0.5 mM scopoletin solution containing 10 µM ferritin (0 V for 1 s, 0.9 V for 0.1 s, 100 cycles).

Over time, sensor technology using screen-printed electrodes has found its potential in various applications. It is known for its capacity to enable fast, accurate, and portable analyses at low cost [40,41]. Figure 1 presents the formation of an MIP layer on an SPC electrode by the electropolymerization of scopoletin in the presence of ferritin through multistep amperometry, involving 100 cycles, in which the SPC electrode was polarized at 0 V for 1 s and at 0.9 V for 1 s.

**Figure 2:**
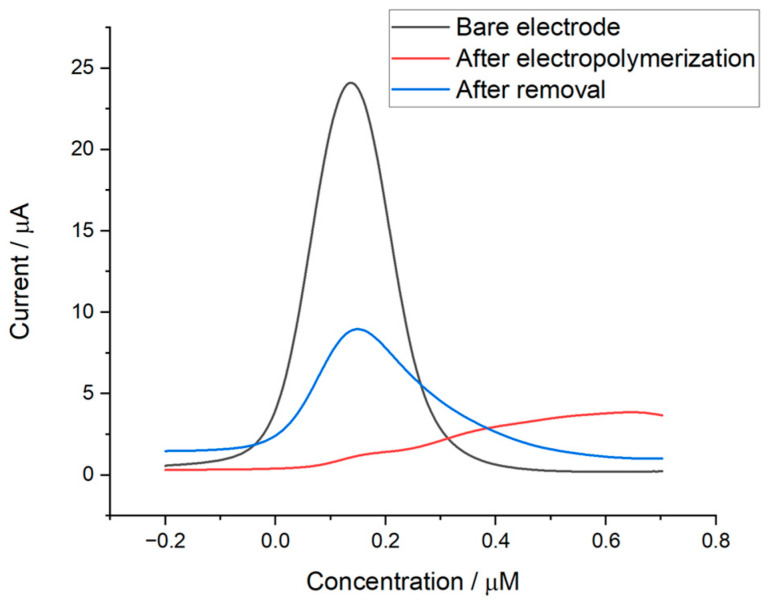
DPVs of SPC electrodes in 5 mM ferricyanide/ferrocyanide solution (in 100 mM KCl): bare electrode (black), after electropolymerization (red), and after electrochemical removal from HEPES buffer (2 × 120; blue).

The predominant method used for the characterization of electrochemical MIP sensors evaluates the diffusional permeability of the polymer layer to a redox marker, such as ferricyanide/ferrocyanide, using cyclic voltammetry, differential pulse voltammetry, square wave voltammetry, or electrochemical impedance spectroscopy (as depicted in Figure 2) [1]. As demonstrated in Figure 2, a non-conducting film was formed after electropolymerization (red curve).

A pivotal stage in the fabrication of sensitive and selective MIP sensors is the removal step. Preferably, mild procedures are used to avoid the destruction of the polymeric film. Several approaches have been applied to remove the template ferritin from an MIP-covered SPC electrode. An increase in ionic strength by incubation in 3 M KCl had no effect, while electrochemical treatment in HEPES buffer caused partial removal of template molecules. Nevertheless, the rebinding of ferritin was not achieved. Milder acidic conditions, such as incubation in 50 mM glycine–HCl buffer at pH 2.2, or harsher acidic conditions, such as incubation in 0.1 M H_2_SO_4_, also had a similar effect. In our previous work involving an MIP-covered gold SPR chip, the template ferritin was successfully removed from 1 mM NaOH solution, enabling the rebinding of ferritin [38]. However, the use of three different concentrations of NaOH solution (0.5 mM, 1 mM, and 5 mM) failed to achieve template removal.

#### 3.1.2. Single-Walled-Carbon-Nanotube-Modified Screen-Printed Carbon Electrodes

**Figure 3:**
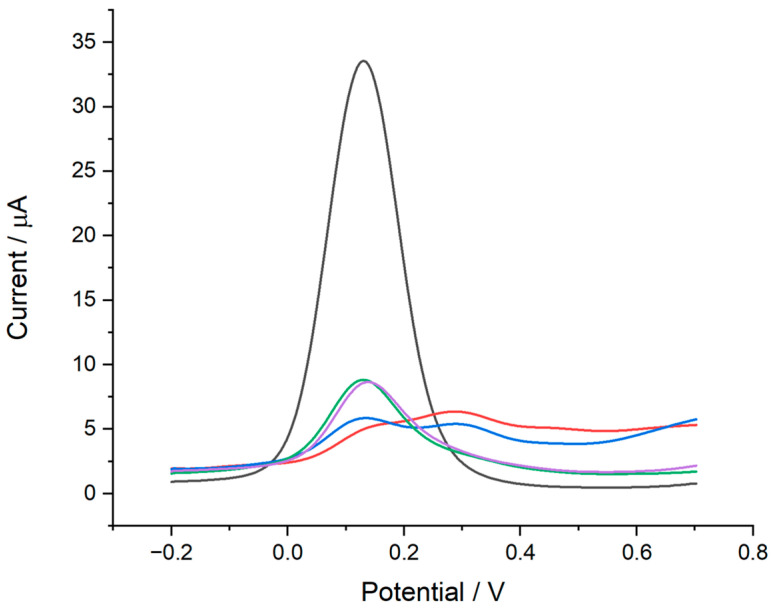
DPVs of SWSP electrodes in 5 mM ferricyanide/ferrocyanide solution (in 100 mM KCl): bare electrode (black), after electropolymerization (red), after overnight incubation in glycine–HCl buffer (50 mM, pH 2.2) (blue), after electrochemical removal from glycine–HCl buffer (50 mM, pH 2.2) using cyclic voltammetry within the potential range of −0.2 V to 1.0 V (50 scans) (green), and after 1 µM ferritin binding for 1 h (purple).

Carbon nanotubes are frequently used in bio(mimetic) sensors due to their ability to increase the electrochemical active surface area and improve electrochemical properties [42,43,44]. In this study, SWSP electrodes were used for MIP sensor preparation. The multistep amperometry method described earlier was used. Following electropolymerization, a non-conducting film was formed (Figure 3). Partial removal of template molecules was achieved in 50 mM glycine–HCl buffer using cyclic voltammetry within the potential range of −0.2 V to 1.0 V (50 scans), whereas incubation in the same buffer alone had no effect. Despite these efforts, successful rebinding was not achieved. Additionally, various other template removal procedures were attempted, with no favorable outcomes. Consequently, further investigation was conducted using a glassy carbon electrode.

### 3.2. Glassy Carbon Electrodes

Glassy carbon electrodes have been widely used in electrochemical biosensing, including MIP-based sensors, owing to their good chemical stability, electrical conductivity, biocompatibility, and cost-effectiveness [45,46]. Furthermore, the integration of modifiers and nanomaterials has been observed to enhance the performance of these sensors.

**Figure 4:**
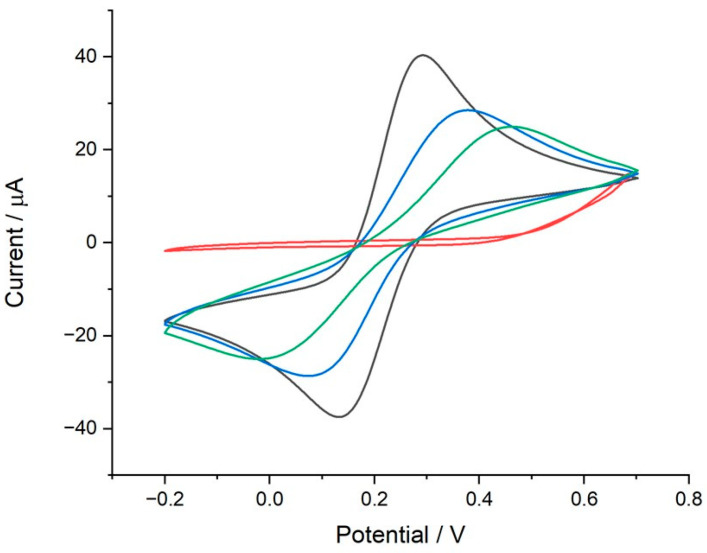
CVs of GCEs in 5 mM ferricyanide/ferrocyanide solution (in 100 mM KCl): bare electrode (black), after electropolymerization (red), after overnight incubation in 5 mM NaOH (blue), and after 0.25 µM ferritin binding for 1 h (green).

The multistep amperometry method described earlier was used. depicted in Figure 4, the MIP preparation process can be monitored by observing the reduction in the signal intensity of redox markers. In contrast to screen-printed electrodes, ferritin molecules can be effectively removed using 5 mM NaOH solution (blue). Furthermore, ferritin molecules can be rebound, reaching equilibration within an hour (green).

**Figure 5:**
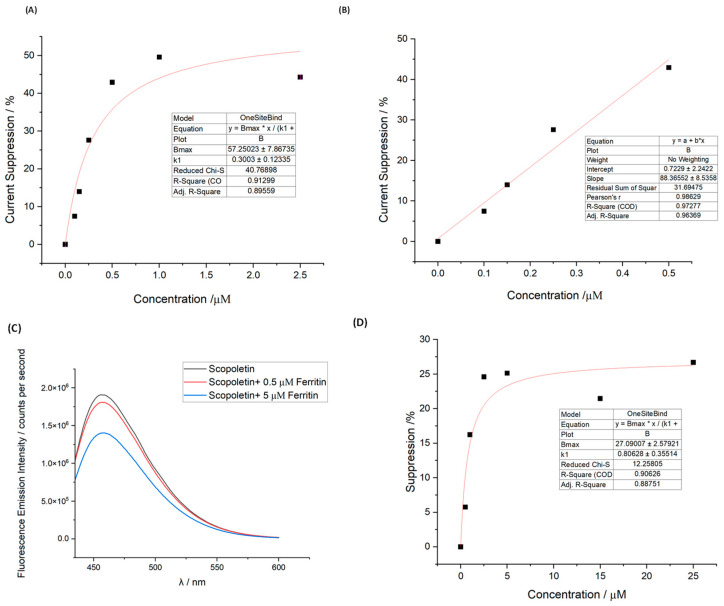
(**A**,**B**) Concentration dependence of relative current decrease for the binding of ferritin to MIP–GCE based on differential pulse voltammetry; (**C**,**D**) fluorescence titration of scopoletin (0.5 mM) with ferritin (1 mM).

Rebinding studies were conducted in 10 mM HEPES buffer (pH 7.4) with varying ferritin concentrations. The change in the signal of the redox marker was investigated using differential pulse voltammetry. The ferritin–MIP electrode linearly suppressed the signal of the redox marker up to 0.5 µM with an R^2^ of 0.97277 (Figure 5A,B), consistent with our previous findings and those of several previously published results (Table 1) [38,47,48]. The limit of detection (LOD) was calculated using the equation 3.3 × SD/m (SD: standard deviation of the blank and the slope of the calibration graph) as 0.097 µM.

Using the One-Site-Specific Binding Model, the dissociation constant (K_d_) was calculated to be 0.30 µM. Similar results were also obtained in solution upon the interaction of scopoletin and ferritin using fluorescence spectroscopy (resulting in the quenching of the scopoletin signal), with a calculated K_d_ of 0.81 µM.

Since the cutoff value of ferritin in the serum of healthy persons is at 3 × 10^−11^ M, the sensitivity of the MIP sensor in this study is not sufficient for the lower ferritin range in serum. The sensor presented by Cai et al. claims to have the required concentration [49] However, the reported measuring range in the lower attomolar concentration range is highly questionable. In contrast, the measuring range and K_d_ value in this study are in accordance with other publications and compatible with thermodynamic data [24].

Electrosynthesized MIPs typically exhibit K_d_ values in the range of μM to nM, i.e., the change in free enthalpy is between −30 and −60 KJoule. A few papers claim even lower limits of detection, in the picomolar range, or even lower measuring ranges over more than four decades of target concentration (Table 2). An explanation for the “ultra-sensitivity” may be that the MIP acts as an ion channel sensor with a semi-logarithmic concentration dependence over several orders of magnitude [50].

In addition, the cross-reactivity of the MIP sensor was studied using human serum albumin (HSA). The binding of 1 µM ferritin led to 49.6% suppression, whereas HSA caused 8.6% suppression. This result demonstrates the low cross-reactivity of the MIP toward a non-target protein and is in accordance with reports in the literature. MIPs for macromolecular targets typically exhibit cross-reactivities above 2% versus the most abundant proteins in serum (HSA, IgG, and hemoglobin). While “real samples,” like human serum, contain the relevant protein biomarker, e.g., for cancer, in the sub-nanomolar range, these potentially interfering proteins are in the millimolar concentration range. Therefore, the MIP should be saturated by an excess of the interfering proteins, and dilution should not solve this problem. However, lateral-flow devices with many separation plates could govern the separation effect of the MIP.

## 4. Conclusions

The general concept of increasing the active surface of electrochemical sensors by using electrodes with high roughness or by integrating nanoparticles, nanotubes, and graphene has been widely studied and transferred to MIP sensors. The comparison of the three different MIP-covered carbon electrodes underlines the findings described in the literature that despite identical MIP synthesis, each surface has a specific performance in template removal. For poly(scopoletin)-based MIPs for the huge globular protein ferritin, an increase in the electroactive surface prevents the preparation of effective MIP layers either by blocking template removal or by rebinding. However, the MIP formed on a flat glassy carbon electrode has an analytical performance, i.e., measuring range (up to 0.5 µM and K_d_ 0.30 µM) comparable to that of the MIP on the gold surface of an SPR chip.

## Figures and Tables

**Table 1:** Examples of MIPs for ferritin sensing.

Materials	Method	Linearity Range	Reference
Quantum dot/ferritin-imprinted nanoparticle-modified test strips	Fluorescence spectroscopy	1.0–6.0 µM	[47]
Ferritin-imprinted poly(phenol) film on CNT-array-modified titanium-coated glass substrate	EIS	2 × 10^−17^–1 × 10^−12^ M	[49]
Ferritin-imprinted poly(scopoletin) film on an Au electrode	CV	0.9–8 µM	[48]
Ferritin-imprinted poly(scopoletin) film on an Au chip	SPR	up to 0.5 µM	[38]
Ferritin-imprinted poly(scopoletin) film on a GCE	DPV	up to 0.5 µM	This work

SPR: surface plasmon resonance; DPV: differential pulse voltammetry; GCE: glassy carbon electrode.

**Table 2:** Electrosynthesized MIPs for proteins with redox markers.

Template	Monomer	Electrode	Linearity Range	K_d_	Reference
Transferrin	Scopoletin	Au	0.1–1 µM	0.56 µM	[32]
Hemoglobin	NHMA	SPE	0.1–1 nM	0.86 ± 0.11 nM	[51]
Lysozyme	Scopoletin	Au	150 nM–20 µM (MIP 2)	-	[15]
IgG	Dopamine	SAM-modified QCM chip	-	296 nM	[52]
Cardiac troponin I	*o*-AP	GCE	0.05–5.0 nM	-	[53]
p-Tau-441	AMP	SPE	2.18 pM–2.18 nM	-	[54]
Annexin A3	Caffeic acid	SPE	2.8 pM–5.5 nM	-	[55]
Troponin T	Pyrrole	SPE	0.26–2.6 pM	0.7 pM	[56]

NHMA: N-hydroxymethylacrylamide; SPE: screen-printed electrode; QCM: quartz crystal microbalance; SAM: self-assembled monolayer; MIP 2: molecularly imprinted polymer (two different methods have been applied for MIP synthesis); GCE: glassy carbon electrode; *o*-PD: *o*-phenylenediamine; *o*-AP: *o*-aminophenol; AMP: 3-aminophenol.

## Data Availability

Data are contained within the article.
